# Circulating tumor DNA as a marker of molecular residual disease in resected esophageal squamous cell carcinoma

**DOI:** 10.1186/s43556-025-00310-6

**Published:** 2025-09-18

**Authors:** Cai-Yan Fang, Jing Wen, Jia-Di Wu, Zhi-Chao Li, Sheng Huang, Yan Huang, Ji-Yang Chen, Hui-Lin Su, Xiu-Ying Xie, Kong-Jia Luo, Jian-Hua Fu, Hong Yang

**Affiliations:** 1 State Key Laboratory of Oncology in South China, Guangdong Provincial Clinical Research Center for Cancer, and, Department of Thoracic Surgery , Sun Yat-Sen University Cancer Center, Guangzhou, 510060 People’s Republic of China; 2 Department of Thoracic Surgery, Sun Yat-Sen University Cancer Center, 651 Dongfeng East Road, Guangzhou, 510060 People’s Republic of China

**Keywords:** Esophageal squamous cell carcinoma, Circulating tumor DNA, Molecular residual disease, Biomarker, Circulating cell-free DNA

## Abstract

Esophageal squamous cell carcinoma (ESCC) remains a major contributor to cancer-related mortality, with molecular residual disease (MRD) detection posing a significant challenge in post-surgical management. This study aimed to evaluate the effectiveness of circulating tumor DNA (ctDNA) in detecting MRD in patients with resectable ESCC undergoing radical surgery. A total of 62 primary tumor tissues, 108 preoperative, and 125 postoperative plasma samples were collected from 125 such ESCC patients who underwent radical surgery, and subjected to sequencing. Next-Generation Sequencing and ultra-high sensitivity Automated Triple Groom Sequencing panels were used to sequence genomic DNA from tumor tissues and ctDNA from plasma, respectively. ctDNA positive mutations included tumor-informed mutations and tumor-naïve mutations. Key findings revealed a high concordance rate of mutation detection between primary tumor tissue and preoperative plasma samples (91.11%, *p* = 0.62). Critically**,** the recurrence rate was higher in postoperative ctDNA-positive ESCCs than that in negative ones (66.67% (40/60) vs. 21.54% (14/65), *p* < 0.001). And postoperative ctDNA-positivity was associated with poorer disease-free survival (DFS) (hazard ratio (HR): 4.58, 95% CI: 2.65–7.92, *p* < 0.001) and overall survival (OS) (HR: 5.39, 95% CI: 2.96–9.80, *p* < 0.001). Similar prognostic patterns were observed in patients with preoperative ctDNA-positivity (*p* = 0.014; *p* = 0.016; *p* = 0.071) and ctDNA-nonclearance (*p* < 0.001; *p* < 0.001; *p* < 0.001). Furthermore, in combination with postoperative ctDNA status, the Tumor-Node-Metastasis-Blood (TNMB) staging system was able to better distinguish patients with different prognoses compared with traditional TNM (*p* < 0.001). In conclusion, postoperative ctDNA-positivity emerges as a promising biomarker for detecting MRD in ESCC patients following surgical resection.

**Supplementary Information:**

The online version contains supplementary material available at 10.1186/s43556-025-00310-6.

## Introduction

In 2022, esophageal cancer (EC) was the 11th most diagnosed cancer globally (511,000 cases) and the 7th deadliest (445,000 deaths)[1]. China is a country with a high incidence of EC, accounting for half of these cases and deaths[2], predominantly esophageal squamous cell carcinoma (ESCC) (~ 90%). Surgery remains the cornerstone in treatment for resectable ESCCs, but over half of the patients face recurrence and metastasis post-surgery[3], resulting in a low 5-year survival rate of 25%[4]. Research indicates that after treatment, certain patients exhibit molecular residual diseases (MRDs) that persist undetected through imaging or standard examinations, potentially leading to disease relapse or metastasis [5, 6]. At the point when a solid tumor becomes detectable for recurrence or metastasis via conventional methods, the cancer cell count typically exceeds tens of millions, rendering eradication highly unlikely, irrespective of the available treatment modalities. Consequently, the identification of MRD holds substantial clinical significance for early assessment of therapeutic response, improving prognostic accuracy or risk stratification, and enabling post-treatment surveillance to detect potential relapse and guide proactive intervention.

In general, the detection of MRD depends on the utilization of molecular assays that exhibit exceptional sensitivity and specificity, such as polymerase chain reaction (PCR)-based and next-generation sequencing (NGS)-based technologies. Circulating tumor DNA (ctDNA) is a form of circulating cell-free DNA (cfDNA) that tumor cells release during apoptosis or necrosis[7–10]. Thus, ctDNA can minor the mutation information of tumor DNA in the tumor tissue and can be collected noninvasively. Moreover, Its short half-life (< 2 h) makes ctDNA ideal for real-time monitoring of tumor changes[11, 12].

Recent advancements in ctDNA examination in peripheral blood highlight its potential as a universal biomarker for MRD in solid tumors[13–19]. Colorectal cancer is at the forefront of solid tumor MRD research. The National Comprehensive Cancer Network (NCCN) guidelines for colon cancer now include ctDNA-based MRD assessment, using postoperative ctDNA to identify higher recurrence risk in stage I-III colon cancer[15]. However, ctDNA use for MRD in ESCC is less explored[20–25], with most studies focusing on esophageal adenocarcinoma (EAC)[25–34]. However, earlier research has indicated that squamous cell carcinomas release more ctDNA compared to adenocarcinomas. [25, 35–37].

This study hypothesized that ctDNA-based MRD detection could serve as a robust prognostic biomarker for resected ESCC patients. To evaluate this, we performed high-depth sequencing on 62 primary tumor tissues, 108 preoperative and 125 postoperative plasma samples from 125 surgery-only ESCC patients using targeted NGS panel (437 genes) and ultra-high sensitivity Automated Triple Groom Sequencing (ATG-seq) panel (196 genes). Our findings establish postoperative ctDNA-positivity as both a promising biomarker for MRD detection and an effective tool for risk stratification in resected ESCC patients**,** offering clinical insights to future adjuvant therapy decisions following curative resection.

## Results

### Patient characteristics and sample quality control

In this study, a total of 62 primary tumor tissues, 108 preoperative and 125 postoperative plasma samples were collected from 125 ESCC patients at pathological stages of II-IVA (the 8th edition of the American Joint Committee on Cancer staging system) who underwent radical surgery without neoadjuvant and adjuvant therapy, and subjected to sequencing. The primary tumor tissue and preoperative plasma served as the baseline sample; therefore, all 125 patients had both postoperative plasma and a corresponding baseline sample (i.e., either primary tumor tissue or preoperative plasma) (Fig. S1a). The median follow-up time for the entire cohort was 40.03 months (95% CI: 38.09–41.66). Patient demographics and baseline clinical characteristics are stratified by disease-free survival (DFS) status and presented in Table 1. All samples used passed quality control (Fig. S2), with Q20 scores ≥ 90% and 20X coverage ≥ 90%. The median sequencing depths were 266X for blood control, 981X for tumor tissue, 29,495X for preoperative plasma, and 30,089X for postoperative plasma samples.

**Table 1:** Baseline characteristics

Characteristics	No. of With DFS Events n (%)	No. of Without DFS Events n (%)	No. of Total Patients n
patients	54	71	125
Median age, years (range)	66(45–78)	63(41–82)	63(41–82)
Sex			
Female	9(30.00%)	21(70.00%)	30
Male	45(47.37%)	50(52.63%)	95
Primary tumor location			
Proximal third	3(60.00%)	2(40.00%)	5
Middle third	26(42.62%)	35(57.38%)	61
Distal third	22(43.14%)	29(56.86%)	51
Gastroesophageal junction	0(00.00%)	2(100.00%)	2
Multiple primary	3(50.00%)	3(50.00%)	6
Vascular invasion			
Yes	20(51.28%)	19(48.72%)	39
No	34(39.53%)	52(60.47%)	86
Nerve tract invasion			
Yes	27(46.55%)	31(53.45%)	58
No	27(40.30%)	40(59.70%)	67
Pathological stage			
II	22(32.35%)	46(67.65%)	68
III	25(52.08%)	23(47.92%)	48
IVA	7(77.78%)	2(22.22%)	9

*DFS* disease-free survival

### Mutation profile in tumor tissue and plasma

The mutation detection rates for tumor tissue, preoperative plasma, and postoperative plasma were 98.39% (61/62), 79.63% (86/108), and 48.00% (60/125), respectively (Fig. S1b). Our assay demonstrated a limit of detection (LOD) of 0.01% for variant allele frequency (VAF). Quantitative mutational features, including total mutation count, unique gene involvement, median mutation count (with range), and mean VAF values, are detailed in Table S1.

**Fig. 1:**
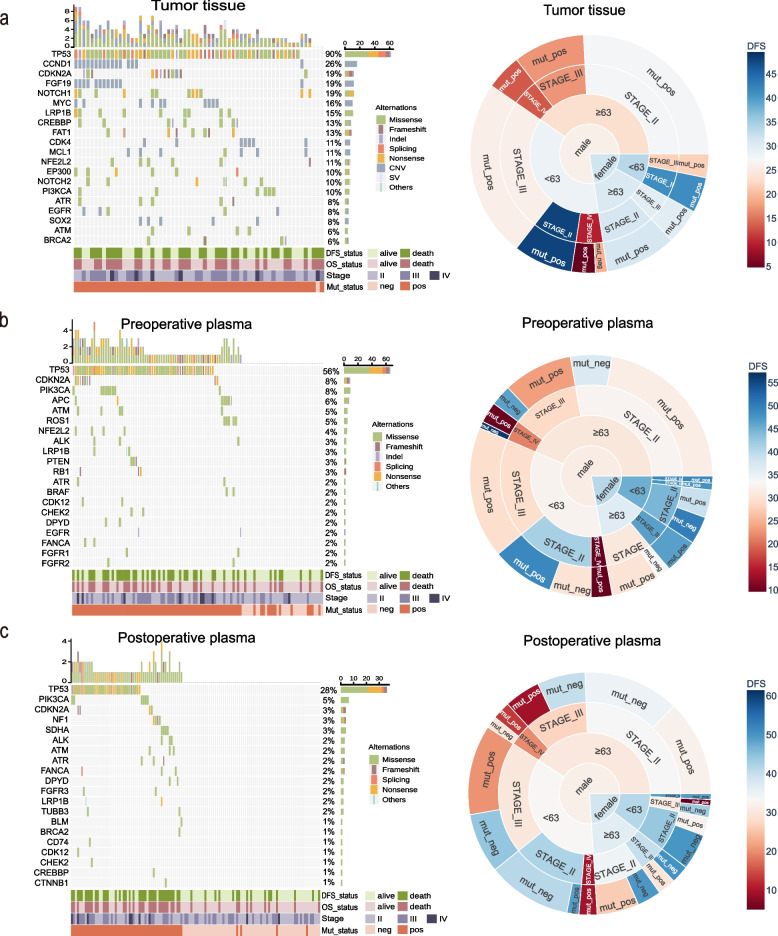
Mutation profiling **a-c.** Mutation landscapes and hierarchical patient data are shown for 62 tumor tissues (**a**), 108 preoperative (**b**) and 125 postoperative (**c**) plasma samples. Hierarchical patient data are presented as sunburst diagrams, with rings representing gender, age, pTNM stage, and mutation status from center outward. Color intensity reflects the mean DFS duration for each subgroup. DFS, disease-free survival

The mutation spectra of the top 20 high-frequency mutated genes and corresponding sunburst diagrams from tumor tissues (Fig. 1a), preoperative plasma (Fig. 1b), and postoperative plasma (Fig. 1c) samples are provided. High-frequency mutation genes detected in the three sample types were consistent with previous literature[21, 38], such as *TP53, CCND1, PIK3CA, CDKN2A, ROS1,* and *ALK* etc. *TP53* had the highest frequency of mutations in tumor tissue, preoperative plasma, and postoperative plasma samples, with rates of 90.3%, 56.5%, and 28.0%, respectively. Pathway enrichment analysis was performed on high-frequency mutated genes (Fig. S3a-c), revealing that the majority of these genes across all three sample types were enriched in four key pathways: the cell cycle, cellular senescence, the p53 signaling pathway, and the FoxO signaling pathway. Mutation genes associated with poor DFS or overall survival (OS) were identified in tumor tissues (Table S2), preoperative plasma (Table S3), and postoperative plasma (Table S4) samples. Thirteen specific gene mutations were significantly associated with inferior survival. The overlapping mutated genes across these sample types were visualized using an UpSet plot (Fig. S3d). Specifically, *TP53* mutations in either preoperative or postoperative plasma samples correlated with worse DFS (*p* = 0.004; *p* = 0.0049) and OS (*p* = 0.037; *p* = 0.0086). Additionally, *SDHA* mutations in postoperative plasma were linked to poorer DFS (*p* = 0.02) and OS (*p* = 0.0049), whereas in preoperative plasma, they were only associated with worse OS (*p* = 0.021). Sunburst plots (Fig. 1a-c) illustrated the stratified distribution of demographic and clinicopathological characteristics and corresponding mean DFS across the three sample cohorts. The analysis revealed a male predominance in patient distribution, with a balanced representation between older (≥ 63 years) and younger (< 63 years) individuals. Additionally, the proportion of patients gradually decreased with advancing pTNM stage (II to IV), while the mutation-positive rates aligned with the aforementioned detection rates.

### Comparison of genomic DNA from tumor tissue to ctDNA from plasma

To evaluate the mutation detection concordance between different sample types, McNemar’s test for paired samples was first applied to assess statistical consistency, followed by percent agreement and prevalence-adjusted bias-adjusted kappa (PABAK) to quantify the agreement level (Table 2). The concordance analysis revealed 91.11% agreement (*p* = 0.62) between tumor tissue and preoperative plasma samples in 45 patients (Fig. S1c), with a PABAK of 0.82 indicating 'almost perfect' agreement according to Landis and Koch criteria [39]. Lower agreement rates were observed in other comparisons: 67.74% (*p* < 0.001) between tumor tissue and postoperative plasma samples (*n* = 62), and 62.96% (*p* < 0.001) between preoperative and postoperative plasma samples (*n* = 108).

**Table 2:** Concordance rate of mutation detection

Preoperative plasma	Tumor tissue		p*	Percent agreement	PABAK
	Negative	Positive			
Negative	0	3	0.62	91.11% (41/45)	0.82
Positive	1	41			
Postoperative plasma	Tumor tissue		p*	Percent agreement	
	Negative	Positive			
Negative	0	19	< 0.001	67.74% (42/62)	
Positive	1	42			
Postoperative plasma	Preoperative plasma		p*	Percent agreement	
	Negative	Positive			
Negative	21	39	< 0.001	62.96% (68/108)	
Positive	1	47			

^*^McNemar's Chi-squared test with continuity correction, a non-significant result (*p* > 0.05) indicates that both methods have equivalent detection rates at the α = 0.05 significance level; Percent agreement, calculated as the proportion of concordant pairs among all paired samples (n_concordant/n_total); PABAK (prevalence-adjusted bias-adjusted kappa), derived as PABAK = 2 × (Percent agreement) − 1, where Percent agreement is expressed as a decimal

**Fig. 2:**
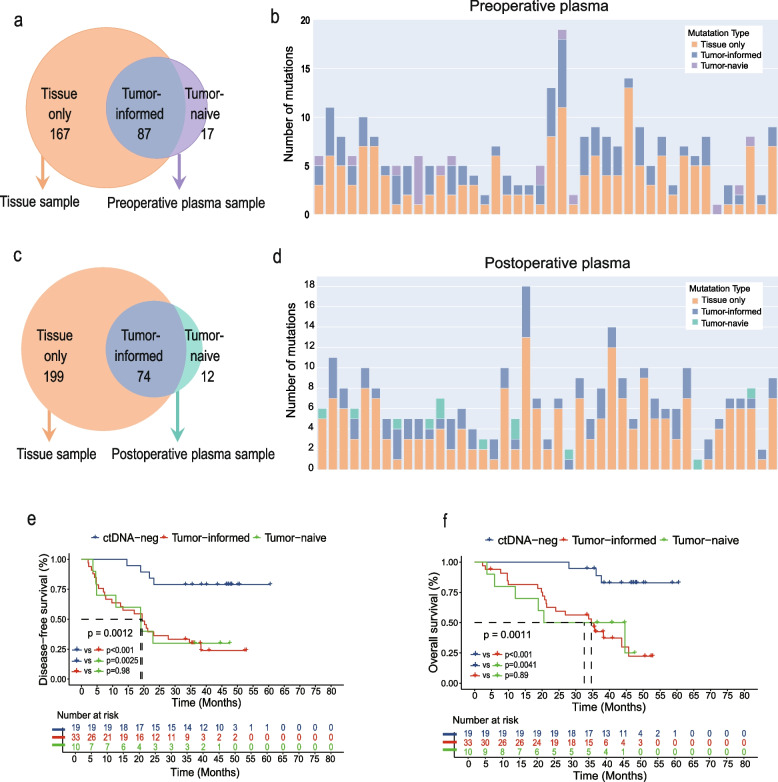
Comparison of genomic DNA from tumor tissue and ctDNA from plasma **a-b**. The number of mutations discovered in 42 paired tumor tissue samples and preoperative plasma samples is presented by a Venn diagram (**a**) or by cases (**b**). **c-d**. The number of mutations discovered in 43 paired tumor tissue samples and postoperative plasma samples is presented by a Venn diagram (**c**) or by cases (**d**). **e–f**. Kaplan–Meier survival curves for DFS (**e**), and Kaplan–Meier survival curves for OS (**f**) among patients with tumor-informed, tumor-naïve, and negative ctDNA mutations. DFS, disease-free survival; OS, overall survival

There were two distinct sources of mutations in ctDNA present in plasma: originating from the tracked mutations derived from tumor tissue, i.e., tumor-informed mutations; and unique variations solely detected in plasma, i.e., tumor-naive mutations. In order to clarify the sources of mutations in ctDNA, we compared the mutations detected in ctDNA-positivity plasma samples and their corresponding paired tumor tissues. Among the preoperative ctDNA-positivity plasma samples, 42 had paired tumor tissue samples that were sequenced (Fig. S1c, Fig. 2a-b). In the 42 paired samples, 254 mutations involving 131 genes were identified in tumor tissue samples, and 104 mutations involving 41 genes in paired preoperative plasma samples. And for the 42 preoperative plasma samples, a total of 87 tumor-informed mutations were detected, while 17 tumor-naive mutations were found. Among the postoperative ctDNA-positivity plasma samples, 43 had paired tumor tissue samples that were sequenced (Fig. S1d, Fig. 2c-d). In the 43 pairs of samples, 273 mutations involving 137 genes were identified in tumor tissue samples, and 86 mutations involving 39 genes in postoperative plasma samples. And for the 43 postoperative plasma samples, 74 tumor-informed mutations were detected, while 12 tumor-naive mutations were found. Among the 45 patients with matched tumor tissue, preoperative plasma, and postoperative plasma sequencing data, the two mutation sources across all three sample types using Venn diagrams were characterized (Fig. S4a).

Next, we explored survival differences among patients with tumor-informed, tumor-naïve, and negative ctDNA mutations. In the 62 patients with paired tumor tissue samples and postoperative plasma samples sequenced (Fig. S1d), 19 were ctDNA-negative, while 43 were ctDNA-positive. Of the ctDNA-positive patients (mutation details in Fig. 2d), 10 harbored tumor-naïve mutations (tumor-naïve group), whereas the remaining 33 exhibited only tumor-informed mutations (tumor-informed group). Patients in the tumor-informed (DFS: hazard ratio [HR] 5.59, 95% CI 2.66–11.73, *p* < 0.001; OS: HR 6.96, 95% CI 3.13–15.49, *p* < 0.001) and tumor-naïve (DFS: HR 5.34, 95% CI 1.36–21.03, *p* = 0.0025; OS: HR 5.89, 95% CI 1.31–26.42, *p* = 0.0041) groups had poorer DFS and OS than those in the ctDNA-negative group. However, no significant difference was observed in DFS (*p* = 0.98) and OS (*p* = 0.89) between patients in the tumor-informed and tumor-naïve groups (Fig. 2e-f). In consideration of the same prognosis of tumor-informed and tumor-naïve ctDNA mutation, we defined ctDNA-positivity as either a tumor-informed or a tumor-naïve ctDNA mutation detected.

### Correlation between ctDNA detection and pathological stage

**Fig. 3:**
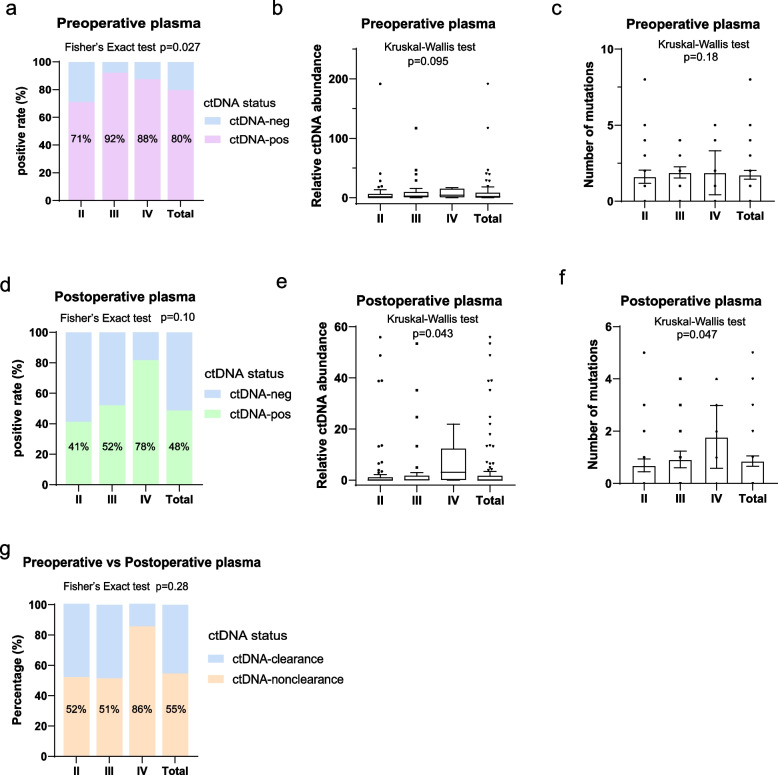
Correlation between ctDNA detection and pathological stage **a-c.** The mutation detection rate (**a**), relative abundance (**b**) and number of mutations of ctDNA (**c**) from preoperative plasma in patients with different pathological stages were compared. **d-f.** The mutation detection rate (**d**), relative abundance (**e**) and number of mutations of ctDNA (**f**) from postoperative plasma in patients with different pathological stages were compared. **g.** The mutation clearance rate in patients with different pathological stages was compared. ctDNA-neg, ctDNA-negativity; ctDNA-pos, ctDNA-positivity. Fig. b and e are Tukey box plots. Fig. c and f are bar plots with error bars (mean ± 95% CI)

An increase in preoperative ctDNA-positivity rates was observed with increasing pathological stage, with rates of 70.97% (44/62), 92.11% (35/38), and 87.50% (7/8) for pathological stage II, III, and IVA patients, respectively (*p* = 0.027) (Fig. 3a). Numerical but not significant differences in the relative abundance of mutations (quantified as maxVAF × cfDNA concentration) and the number of mutations of ctDNA from preoperative plasma were identified in patients with different pathological stages (Fig. 3b-c). In terms of postoperative plasma, there was an increase in the detection rate of ctDNA mutations as overall pathological stage increased. Rates were 41.18% (28/68), 52.08% (25/48), and 77.78% (7/9) for patients with pathological stages II, III, and IVA, respectively (*p* = 0.10) (Fig. 3d). Similarly, as stage advanced, both the relative abundance (*p* = 0.043) and number of mutations (*p* = 0.047) in ctDNA from postoperative plasma increased (Fig. 3e-f).

We also evaluated the conversion patterns of ctDNA detection status in 108 patients with matched pre- and postoperative plasma samples (Fig. S1e). Among 22 preoperative ctDNA-negative patients, 21 (95.5%) showed persistent negativity, whereas 1 (4.5%) developed ctDNA-positivity postoperatively. The single ctDNA-positive patient demonstrated worse median DFS (7.76 vs not reached [NR] months, *p* = 0.0023) and OS (21.4 vs NR months, *p* = 0.0031) compared to persistent negativity patients. Of 86 preoperatively ctDNA-positive patients, 39 (45.4%) converted to negative status (ctDNA-clearance) and 47 (54.6%) remained positive (ctDNA-nonclearance). The ctDNA clearance rate (ctDNA-clearance/[ctDNA-clearance + ctDNA-nonclearance]) showed a decreasing trend with advancing pathological stage: 47.73% (21/44) in stage II, 48.57% (17/35) in stage III, and 14.29% (1/7) in stage IVA patients (*p* = 0.28) (Fig. 3g).

### Correlation between ctDNA detection status and risk of recurrence or death

**Fig. 4:**
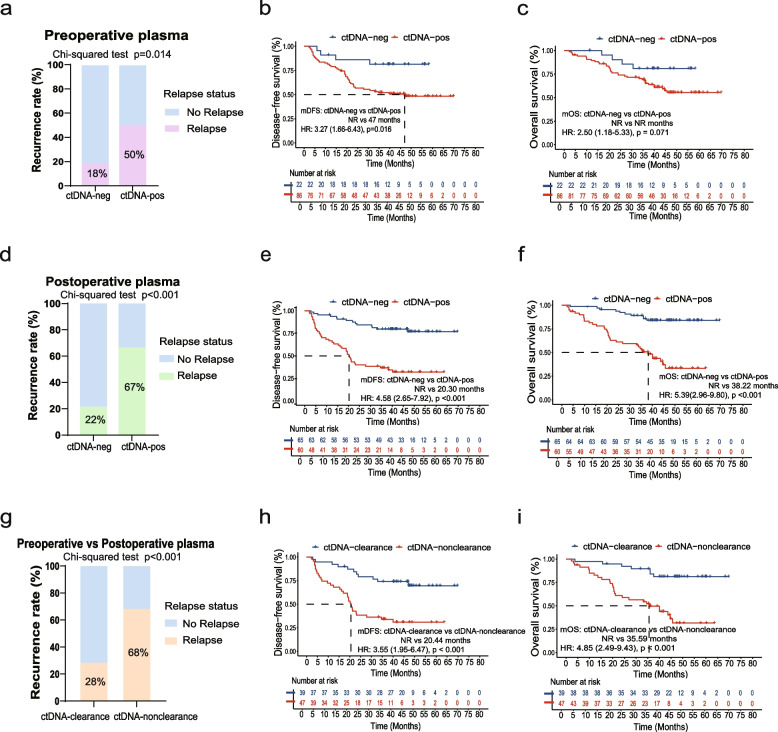
Correlation between ctDNA detection status and risk of recurrence or death **a-c.** Overall relapse proportion (**a**), Kaplan–Meier survival curves for DFS (**b**), and Kaplan–Meier survival curves for OS (**c**) between patients with ctDNA-negativity and ctDNA-positivity preoperatively. **d-f.** Overall relapse proportion (**d**), Kaplan–Meier survival curves for DFS (**e**), and Kaplan–Meier survival curves for OS (**f**) between patients with ctDNA-negativity and ctDNA-positivity postoperatively. **g-i.** Overall relapse proportion (**g**), Kaplan–Meier survival curves for DFS (**h**), and Kaplan–Meier survival curves for OS (**i**) between patients with ctDNA-clearance and patients with ctDNA-nonclearance. HR, hazard ratio. DFS, disease-free survival; OS, overall survival; ctDNA-neg, ctDNA-negativity; ctDNA-pos, ctDNA-positivity

The recurrence rate of the total cohort was 43.20% (54/125) and an increase in recurrence was observed with pathological stage progression, with rates of 32.35% (22/68), 52.08% (25/48), and 77.78% (7/9) for pathological stage II, III, and IVA patients, respectively (Fisher’s Exact test, *p* = 0.010). For outcomes based on preoperative plasma, patients with preoperative ctDNA-positivity had a higher recurrence rate than those with preoperative ctDNA-negativity (50.0% (43/86) vs. Negativity: 18.18% (4/22), *p* = 0.014) (Fig. 4a). Also, survival analyses revealed a statistically poorer DFS (*p* = 0.016) (Fig. 4b) and a trend toward worse OS (*p* = 0.071) (Fig. 4c) in patients with preoperative ctDNA-positivity.

Notably, patients with postoperative ctDNA-positivity had a higher recurrence rate than those with ctDNA-negativity (66.67% (40/60) vs. 21.54% (14/65), *p* < 0.001) (Fig. 4d). Moreover, DFS was significantly worse in patients with postoperative ctDNA-positivity (DFS: 20.30 vs. NR months, HR: 4.58, 95% CI: 2.65–7.92, *p* < 0.001) (Fig. 4e), as was their OS (mOS: 38.22 vs. NR months, HR: 5.39, 95% CI: 2.96–9.80, *p* < 0.001) (Fig. 4f). A higher recurrence risk (Positivity: 68.09% (32/47) vs. Negativity: 28.21% (11/39), *p* < 0.001) (Fig. 4g), as well as worse DFS (mDFS: 20.44 vs. NR months, HR: 3.55, 95% CI: 1.95–6.47, *p* < 0.001) (Fig. 4h) and OS (mOS: 35.39 vs. NR months, HR: 4.85, 95% CI: 2.49–9.43, *p* < 0.001) (Fig. 4i) were also observed in patients without ctDNA-clearance after surgery.

**Fig. 5:**
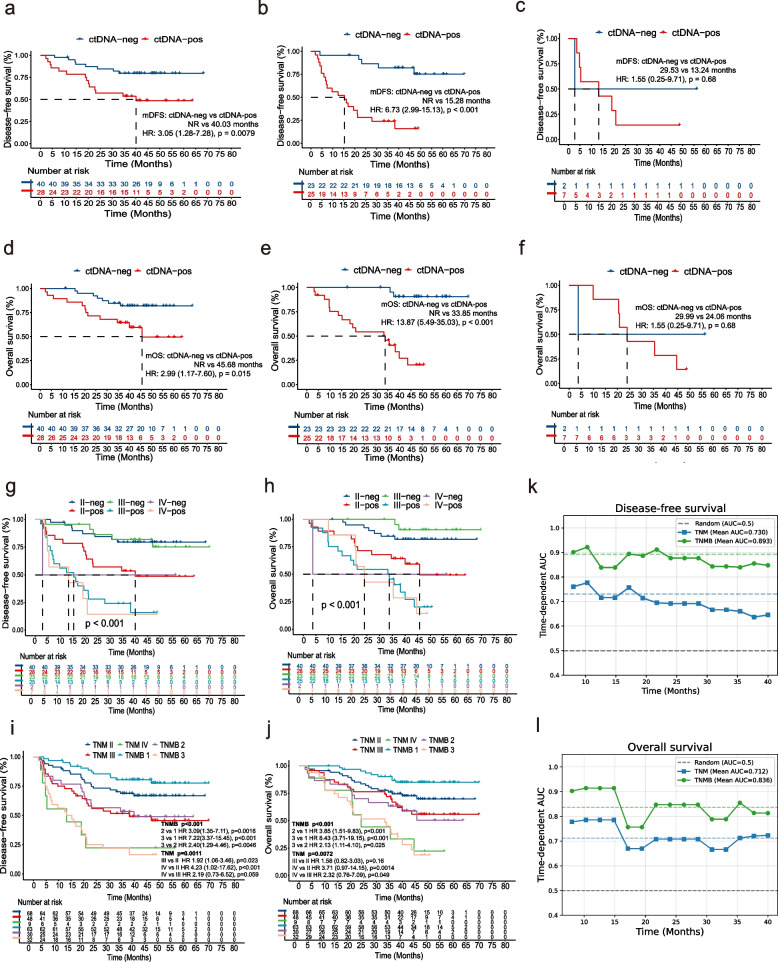
Subgroup analysis and TNMB staging system **a-c.** Kaplan–Meier survival curves for DFS in patients with pathological stage II (**a**), stage III (**b**), and stage IVA (**c**). **d-f.** Kaplan–Meier survival curves for OS in patients with pathological stage II (**d**), stage III (**e**), and stage IVA (**f**). **g-h**. Exploratory Kaplan–Meier analysis for DFS (**g**) and OS (**h**) among patients in group II-neg, group II-pos, group III-neg, group III-pos, group IVA-neg, and group IVA-pos. **i-j.** The integration of Kaplan–Meier analysis for DFS (**i**) and OS (**j**) in patients divided by TNM staging and the TNMB staging system. **k-l** (**k**) DFS and (**l**) OS time-dependent ROC comparing TNM (blue line) vs. TNMB (green line) staging system. neg, ctDNA-negativity; pos, ctDNA-positivity; TNM, Tumor-Node-Metastasis; TNMB, Tumor-Node-Metastasis-Blood; DFS, disease-free survival; OS, overall survival; ROC, receiver operating characteristic; AUC, area under the receiver operating characteristic curve

The results of multivariate Cox regression analysis reinforced the potential of postoperative ctDNA-positivity as a reliable indicator for MRD, as it was identified as an independent prognostic factor for DFS (HR: 4.10, 95% CI: 2.03–8.29, *p* < 0.001) (Table 3) and OS (HR: 5.38, 95% CI: 2.65–10.95, *p* < 0.001) (Table 3). The prognostic value of postoperative ctDNA-positivity was also explored in different subgroups (Fig. 5a-f). Compared with postoperative ctDNA-negative patients, pathological stage II and III patients with postoperative ctDNA-positivity all had a worse prognosis, including lower DFS (stage II: *p* = 0.0079; stage III: *p* < 0.001) (Fig. 5a-b) and OS (stage II: *p* = 0.015; stage III: *p* < 0.001) (Fig. 5d-e). There were a few patients with stage IVA disease, and no significant differences in DFS (*p* = 0.68) and OS (*p* = 0.68) were detected for stage IVA patients (Fig. 5c, f).

**Table 3:** DFS and OS analysis of clinicopathologic variables and ctDNA status in ESCC

Variables	No. of patients	Univariate analysis		Multivariate analysis	
		**HR (95% CI)**	**p**	**HR (95% CI)**	**p**
**DFS**					
Sex (male vs. female)	95/125	1.85(0.91–3.79)	0.091		
Age (≥ 63 vs. < 63)	68/125	1.19(0.69–2.04)	0.53		
Stage III-IVA vs. Stage II	57/125	2.17(1.26–3.75)	0.0052	1.67(0.93–2.99)	0.084
Nerve tract invasion	58/125	1.20(0.71–2.05)	0.50		
Vascular invasion	39/125	1.44(0.83–2.51)	0.19		
Preoperative ctDNA-positivity	86/108	3.28(1.18–9.13)	0.023	1.28(0.41–4.06)	0.67
Postoperative ctDNA-positivity	60/125	4.72(2.55–8.71)	< 0.001	4.10(2.03–8.29)	< 0.001
**OS**					
Sex (male vs. female)	95/125	2.03(0.90–4.54)	0.086		
Age (≥ 63 vs. < 63)	68/125	1.41(0.77–2.56)	0.26		
Stage III-IVA vs. Stage II	57/125	1.87(1.03–3.37)	0.039	1.69(0.94–3.07)	0.082
Nerve tract invasion	58/125	1.28(0.71–2.29)	0.41		
Vascular invasion	39/125	1.42(0.78–2.59)	0.26		
Preoperative ctDNA-positivity	86/108	2.51(0.89–7.06)	0.082		
Postoperative ctDNA-positivity	60/125	5.55(2.73–11.26)	< 0.001	5.38(2.65–10.95)	< 0.001

*DFS* disease-free survival, *OS* overall survival, *ctDNA* circulating tumor DNA, *ESCC* esophageal squamous cell carcinoma

The efficacy of postoperative ctDNA-positivity for MRD detection was further evaluated. The performance of the postoperative ctDNA assay for recurrence prediction within one year of follow-up was 83.33% sensitivity and 60.40% specificity, with an overall accuracy of 64.8% (81/125) (Table S5). As the follow-up time increased, the sensitivity and negative predictive value (NPV) of postoperative ctDNA positivity for recurrence prediction decreased, but the specificity and positive predictive value (PPV) increased. Relatively high values of sensitivity and NPV for the postoperative ctDNA assay were observed for recurrence prediction within one year, two years, or three years of follow-up.

### Combination of the TNM stage system and ctDNA detection status

Given the above findings, we combined the postoperative ctDNA detection status with the traditional pathological tumor-node-metastasis (TNM) staging to assess patient prognosis, which was defined as tumor-node-metastasis-blood (TNMB) staging. Based on exploratory results (Fig. 5g-h), the specific staging criteria for TNMB were as follows: pathological stage II as well as ctDNA-negativity and pathological stage III as well as ctDNA-negativity were defined as TNMB stage 1; pathological stage II as well as ctDNA-positivity and pathological stage IVA as well as ctDNA-negativity were defined as TNMB stage 2; and pathological stage III as well as ctDNA-positivity and pathological stage IVA as well as ctDNA-positivity were defined as TNMB stage 3. We found that this new TNMB staging system was more effective in distinguishing patient prognosis than the traditional TNM staging (Fisher's Exact Test, *p* < 0.001) (Fig. 5i-j). Time-dependent receiver operating characteristic (ROC) curves demonstrated the discriminative ability of TNMB and TNM staging for DFS and OS at different time points (Fig. 5k-l). TNMB demonstrated consistently higher predictive accuracy than TNM staging, achieving higher C-indices (DFS: 0.80 vs. 0.65; OS: 0.77 vs. 0.69). These findings were further validated in an external cohort[40] of ESCC patients receiving definitive chemoradiotherapy combined with toripalimab (Fig. S4b-c), where TNMB maintained superior prognostic discrimination (PFS: 0.75 vs. 0.71; OS: 0.80 vs. 0.72). The results suggest that the TNMB staging system can enhance and refine traditional TNM staging in predicting patient prognosis.

## Discussion

Our study analyzed gene mutations in 62 primary tumor tissues, 108 preoperative plasma samples, and 125 postoperative samples from 125 ESCC patients who underwent surgery. Using ATG-seq (196 genes) with 50 ng of ctDNA per sample, we achieved a sequencing depth of ~ 30,000 × and a VAF detection Limit of 0.01%. Combining tumor-informed and tumor-naive methods, we found high concordance between tumor tissue and preoperative plasma, as well as high mutation detection rates in both preoperative and postoperative plasma. Preoperative and postoperative ctDNA-positivity, along with ctDNA-nonclearance, were linked to higher recurrence rates and poorer DFS and OS. Postoperative ctDNA-positivity was identified as an independent prognostic indicator for DFS and OS, acting as a dependable biomarker for identifying MRD in ESCC patients.

Our study found postoperative ctDNA testing achieved 83.33% sensitivity and 60.40% specificity for one-year recurrence prediction (64.8% accuracy). Compared to imaging modalities[41, 42] which showed lower sensitivity (N-stage: 35–42%; M-stage: 46–69%) but higher specificity (N-stage: 87–93%; M-stage: 74–93%) with 63–84% accuracy, ctDNA provided superior sensitivity for MRD detection while maintaining acceptable accuracy despite lower specificity. The results further substantiated the utility of postoperative ctDNA testing for MRD detection and its potential to enhance postoperative surveillance protocols. We propose integrating longitudinal plasma ctDNA monitoring into follow-up protocols, where positive results could trigger confirmatory imaging. This approach may enable earlier recurrence detection while reducing unnecessary resource utilization, though further validation is required. Regarding ctDNA-guided adjuvant therapy, our comprehensive literature review reveals that there are no large-scale randomized controlled trials demonstrating overall survival benefits from adjuvant radiotherapy or chemotherapy in ESCC, and these treatments remain non-standard[43, 44]. However, the landmark CheckMate 577 trial[45] revealed that adjuvant nivolumab administered for one year significantly improved DFS in patients with locally advanced esophageal or gastroesophageal junction cancer who achieved R0 resection but had residual disease (non-pathologic complete response) after neoadjuvant chemoradiotherapy. In a phase II clinical trial[46] comparing neoadjuvant PD-L1 inhibitor (socazolimab) plus chemotherapy versus chemotherapy alone in locally advanced ESCC, Yue et al.[47] subsequently performed MRD detection through postoperative cfDNA analysis. Their results showed that among 11 MRD-positive patients, none of the 7 patients who received adjuvant socazolimab experienced disease progression, whereas 3 of the 4 untreated patients developed recurrence during the median follow-up period of 17 months (HR: 0.0032; 95% CI, 0.003–0.390; *p* = 0.007). Moving forward, clinical investigations should evaluate the potential role of postoperative ctDNA status in stratifying patients for adjuvant immunotherapy regimens.

The TNMB staging system extends the traditional TNM framework by incorporating tumor biological behavior and molecular characteristics (B), in addition to primary tumor extent (T), nodal involvement (N), and distant metastasis (M). Building on landmark MRD status assessed via PROPHET, Chen et al.[48] first proposed the TNMB staging system for non-small cell lung cancer and found that TNMB staging showed a better prognostic effect than traditional TNM staging. While the TNMB criteria in our study differ from theirs due to variations in cancer type, our results similarly confirmed that the TNMB system provides significantly better prognostic stratification for ESCC patients (*p* < 0.001). Notably, this new system showed significantly higher predictive accuracy compared to the conventional TNM staging (DFS: 0.80 vs. 0.65; OS: 0.77 vs. 0.69). This improvement likely stems from the ability of postoperative ctDNA-positivity to identify patients with MRD who are resistant to surgical intervention. The underlying rationale parallels that of ypTNM staging, which identifies treatment-resistant patients with residual tumors after neoadjuvant therapy. The ypTNM staging paradigm[49] exhibits globally depressed survival outcomes, with early-stage patients significantly worse than pTNM counterparts while advanced-stage patients show comparable or inferior survival to conventional staging. Enhanced discrimination between prognostic groups enables more precise stratification for adjuvant therapy decision-making, thereby advancing the goals of personalized precision medicine. A review of the existing literature reveals that the TNMB stage system for esophageal cancer has been rarely explored, and its introduction might open up new directions and perspectives for future esophageal cancer research and treatment.

Existing literature [20–25] on ctDNA detection in ESCC patients has utilized fixed panels, with various factors significantly influencing the probability of detecting ctDNA. These elements encompass the ctDNA concentrations in a plasma sample, the sequencing depth at particular mutant sites, and the scope of analysis, which pertains to the number of mutations monitored[50]. Previous studies [21, 22, 37] have reported mutation detection rates of 53.3% ~ 71.7% for preoperative plasma, and 14.2% ~ 15.8% for postoperative plasma in ESCC patients. In our study, the detection rate of ctDNA was relatively high at 79.63% (86/108) in the total population preoperatively and 48.00% (60/125) postoperatively, which might be attributed to several key factors. Firstly, our study achieved a highly sensitive LOD for VAF of 0.01% in ctDNA testing, allowing for the identification of even trace amounts of ctDNA. However, previous studies regarding NGS MRD detection in esophageal cancer have rarely reported LOD or had a LOD of 0.1% ~ 0.2%[23, 24, 27]. Second, our deep sequencing approach (a mean depth of ~ 30,000 ×) provided thorough and in-depth analysis of ctDNA mutations, enhancing our ability to detect and characterize genetic alterations accurately. Nevertheless, previous studies of NGS MRD detection in esophageal cancer have rarely reported sequencing depth or had a relatively low mean sequencing depth of ~ 7000 × [31]. Additionally, utilization of a large NGS panel (437 genes) expanded the scope of genomic alterations that could be captured and analyzed. Last, integration of tumor-informed and tumor-naive methods played a significant role in optimizing ctDNA detection and further improving the overall detection rate in our research. In general, researchers[20–25] employ either tumor-informed or tumor-naive approaches for analyzing MRD. The tumor-informed approach utilizes mutation data from primary tumor tissue, forming a personalized mutation profile for each patient and using it for MRD monitoring. The tumor-naive approach aims to achieve the highest sensitivity by using UMIs, increasing sequencing depth, and employing advanced algorithms, without relying on any information from tumor tissue. This approach solely relies on blood detection to obtain accurate results. Our analysis revealed that mutations identified by both methods correlated with worse DFS and OS, yet relying solely on tumor-informed analysis would have missed these high-risk patients. And integration of these methods can improve the detection of ctDNA mutations, providing valuable insights for MRD detection.

Notably, similar observations have been reported in colorectal cancer. Martínez-Castedo et al.[51] showed that both tumor-informed and tumor-naïve approaches are clinically valuable for assessing postoperative risk and making decisions about adjuvant chemotherapy, emphasizing that although tumor-informed methods currently provide greater accuracy in MRD detection, tumor-naive techniques are becoming more popular because of their ease of use and enhanced performance. In our study, we enhanced the tumor-naïve methodology by integrating UMIs, increasing sequencing depth, and employing advanced bioinformatics algorithms, thereby achieving a detection sensitivity of 0.01% VAF. Our hybrid strategy represents a practical compromise, helping to identify clinically relevant mutations without requiring tumor tissue sequencing, while still maintaining reliable detection performance.

In this study, we designed NGS gene panels as pan-cancer detection tools, encompassing full exonic regions and critical intronic regions of cancer-related genes. The panel includes not only well-characterized esophageal cancer-associated genes[52] (e.g., *TP53, CDKN2A, PIK3CA, NOTCH1, KMT2D*) but also other clinically relevant genes across multiple tumor types, ensuring broad coverage for Liquid biopsy applications. Our analysis revealed 13 specific gene mutations significantly correlated with adverse survival outcomes: *TP53, SDHA, ATR, NFKBIA, FANCI, ATRX, LRP1B, NFE2L2, CDKN2A, TUBB3, ATM, PIK3CA, VHL, and ATR*. Among these, *TP53* and *SDHA* mutations in plasma showed comparatively significant prognostic value. Supporting this finding, Li et al.[53] reported that the *TP53* hotspot mutation p.R213* was found to independently predict lower survival rates, with a hazard ratio of 3.37, even after adjustment for clinical variables. Conversely, the role of *SDHA* mutations in ESCC remains understudied in the literature. Based on these preliminary findings, we suggest considering the inclusion of these candidate genes in future ESCC prognostic panels to enhance prognostic accuracy and clinical utility, pending further validation in larger cohorts.

Most studies [20, 21, 23–25] have seldom shown poorer prognoses in patients with preoperative ctDNA-positivity or persistent ctDNA levels. For instance, Iwaya[20] et al. enrolled 36 ESCC patients and discovered no notable difference in OS between those with ctDNA-positive and ctDNA-negative pretreatment plasma (*p* = 0.46). In our current study, there were 108 patients with preoperative plasma available, and we found that those with preoperative ctDNA-positivity had a higher pathological stage (*p* = 0.027) and recurrence rate (*p* = 0.014), worse DFS (*p* = 0.016) and a trend toward poorer OS (*p* = 0.071). Moreover, compared with preoperative plasma, a notable decrease in the mutation detection rate of ctDNA from postoperative plasma was observed (preoperative: 79.63% vs. postoperative: 48.00%, Chi-squared test, *p* < 0.001), probably caused by the surgery and reduction in tumor burden. Patients with ctDNA-nonclearance also had a higher recurrence rate (*p* < 0.001), and poorer DFS (*p* < 0.001) and OS (*p* < 0.001). To date, there is a scarcity of literature reporting on the clinical significance of ctDNA-nonclearance in patients with ESCC. These results highlight the importance of ctDNA in evaluating tumor load and its potential role as a prognostic marker for informing clinical treatment choices for patients.

In our study, tumor tissue sequencing was not performed for all patients due to practical constraints. Instead, we utilized a combination of tumor tissue (*n* = 62) and preoperative plasma (*n* = 108) sequencing as baseline samples, while postoperative plasma (*n* = 125) sequencing was used to assess MRD. All 125 patients with postoperative plasma data had matched baseline sequencing from either tumor tissue or preoperative plasma. Specifically, among these patients, 45 had both tumor tissue and preoperative plasma sequencing, 17 had only tumor tissue sequencing, and 63 had only preoperative plasma sequencing. This design enabled multiple paired analyses: the 45 patients with both sample types allowed us to evaluate mutation concordance between tissue and preoperative plasma; the 62 patients with tumor tissue data (45 plus 17) supported survival comparisons between tumor-informed, tumor-naïve, and ctDNA-negative subgroups; and the 108 patients with pre- and postoperative plasma samples (45 plus 63) facilitated ctDNA clearance analysis. Although universal tumor sequencing could have increased the statistical power of tissue-based analysis, the primary findings regarding postoperative ctDNA-based MRD detection remain robust, given the high concordance between tumor tissue and preoperative plasma (91.11%) and the comparable survival outcomes in patients with tumor-naïve and tumor-informed mutations. The relatively low concordance rates between preoperative and postoperative plasma (62.96%) and between tumor tissue and postoperative plasma (67.74%) are also noteworthy. These discrepancies may be attributed to reduced tumor burden following surgery, tumor heterogeneity, or tumor evolution. Supporting this observation, in the 45 patients with matched tumor tissue, preoperative, and postoperative samples (Fig. S4a), postoperative plasma acquired 12 additional mutations not detected in tumor tissue and 22 new mutations absent in preoperative plasma.

There are several constraints in our research. Our study was conducted retrospectively, which may introduce bias and limit the strength of our conclusions. Future prospective studies will provide more robust evidence. Additionally, our study was limited to ctDNA testing at a single time point after surgery, without multiple time point sampling. Consequently, we could not assess whether ctDNA can detect MRD earlier than imaging examinations. Lastly, we limited our study to patients who did not receive neoadjuvant or adjuvant therapy (between September 12, 2016, and January 8, 2020), following the treatment guidelines of that time period. As a consequence, we were unable to evaluate the recurrence predictive value of ctDNA for patients with resectable ESCC who received neoadjuvant or adjuvant therapy.

In conclusion, we found that preoperative ctDNA-positivity, postoperative ctDNA-positivity and ctDNA-nonclearance were associated with worse DFS and OS and that postoperative ctDNA was an independent prognostic factor for DFS and OS in ESCC patients after esophagectomy. The TNMB staging system demonstrates greater efficacy in distinguishing patient prognosis than traditional TNM staging. Postoperative ctDNA-positivity can be a potent prognostic biomarker for MRD detection in ESCC.

## Methods

### Study design

Conducted at Sun Yat-sen University Cancer Center, this observational cohort study included 125 patients with resectable ESCC between September 12, 2016, and January 8, 2020. Eligible patients had resectable ESCC with a pathological stage of II-IVA according to the 8th edition of the American Joint Committee on Cancer staging system. All patients underwent radical surgery, consisting of either McKeown or Ivor Lewis esophagectomy, which included a two-field lymphadenectomy with total mediastinal lymph node dissection, and did not receive any neoadjuvant or adjuvant therapy. Formalin-Fixed Paraffin-Embedded (FFPE) tumor tissues and plasma samples (before and 7–14 days post-surgery) were obtained from the Sun Yat-sen University Cancer Center Bio-bank for DNA extraction. Follow-ups were every three months in the first year, then biannually until death. The study received ethics approval by the Ethics Committee of Sun Yat-sen University Cancer Center and exemption from informed consent was granted (Consent Letter Number: B2022-090–01; Consent Date: Jan 21, 2022). The registration number for our study on ClinicalTrials.gov is ChiCTR2400093167.

### Next-gen sequencing applied to genomic DNA and ctDNA

DNA was extracted from FFPE sections and whole blood control samples using the QIAamp DNA FFPE Tissue kit (Qiagen) and the DNeasy Blood and Tissue kit (Qiagen), respectively. The QIAamp Circulating Nucleic Acid Kit (Qiagen) was used to extract plasma ctDNA from every plasma sample. A 437-gene targeted NGS panel from Nanjing Geneseeq Technology Inc. (Table S6) was employed for analyzing tumors and white blood cells, whereas a 196-gene panel (Table S7) was used for plasma ctDNA. The KAPA Hyper Prep kit from KAPA Biosystems was used to prepare sequencing libraries. A customized library preparation using a bi-barcoding system and an ultradeep sequencing method known as ATG-Seq (Nanjing Geneseeq Technology Inc.) was utilized for the ctDNA samples. To reduce errors from PCR, hybridization, damage, sequencing, and contamination, and to prevent mutations from non-tumor sources in ctDNA, we implemented the following procedures: (i) sequencing the ctDNA fragment at an approximate depth of 30,000 ×, resulting in redundant DNA molecules; (ii) utilizing mapping positions and a bi-barcode system to enhance the representation of unique DNA molecules; and (iii) employing a duplex-assisted decoder system designed to filter mapping and sequencing artifacts. Sequencing was carried out on the Illumina HiSeq4000 platform, with subsequent data analysis done as previously detailed [54]. For plasma samples containing up to 50 ng of ctDNA, the LOD for VAF was 0.01%, determined using approximately 30,000 × deep sequencing of DNA mixtures from two reference human DNA samples (NA19240 and NA18535).

### Data processing

Trimmomatic [55] was employed for quality control of FASTQ files, removing leading or trailing bases with low quality (below a score of 20) or N bases. The paired-end reads were subsequently aligned to the human reference genome (build hg19) using the Burrows-Wheeler Aligner (BWA) with specified parameters [56]. Picard was used for PCR deduplication, and GATK3 was utilized for local realignment around indels and recalibration of base quality scores. VarScan2 [57] with default settings was used to identify single-nucleotide variations (SNVs) and insertions/deletions in tumor tissue samples. FACTERA [58] was used with default settings to identify genomic fusions.

For ctDNA samples, single-stranded consensus sequences (SSCSs) were created by gathering all read pairs with identical mapping positions and organizing them into distinct SSCS families sharing the same UMI barcode sequences at both ends. At least two reads were required to support a consensus read. Once the SSCS sequence was constructed, two SSCS read pairs with swapped UMI barcode sequences and identical mapping positions were combined into a single duplex consensus sequence (DCS). Subsequently, a local bioinformatics polishing pipeline was employed to detect somatic variants in ctDNA after excluding germline variants using normal control DNA. Mutations found in the corresponding tumor DNA, supported by at least one unique consensus mutant allele read and meeting the polishing criteria, were considered present. Tumor-informed ctDNA positivity was determined by detecting one or more mutations found in the corresponding tumor samples. ctDNA variants absent in the primary tumor are termed tumor-naive ctDNA positivity if the following strict criteria are satisfied, (i) VAF is at least 0.01%, with supporting reads of 5 or more, and a depth of at least 100x; (ii) absent from our earlier published database of clonal hematopoiesis variants [59]; (iii) VAF is 0% in the matched peripheral blood leukocyte sample.

### Statistical analyses

DFS was calculated from the surgery date to the first confirmed radiographic recurrence, death, or the most recent follow-up. OS was measured from the surgery date to either the date of death or the last follow-up. Categorical variables were analyzed using the Chi-square test and Fisher’s Exact test. The t-test was used to compare normally distributed continuous variables, whereas the rank-sum test was applied to those that were not normally distributed. DFS and OS were estimated using the Kaplan–Meier method, and the log-rank test was used to compare the resulting curves. The influence of different factors on survival was evaluated through univariate and multivariate analyses using the Cox proportional hazards model. An analysis of subgroups was performed to evaluate the prognostic significance of postoperative ctDNA positivity in patients categorized by pathologic TNM stages (stage II, stage III, and stage IVA). Clinical variables that were statistically significant in the univariate analysis were used for multivariate analysis. We transformed the TNMB and TNM staging systems into dummy variables and then compared their prognostic performance using time-dependent ROC curves and Harrell's C-index. All *p*-values were calculated using two-tailed tests, with significance determined at *p* < 0.05. The statistical analysis was conducted using R software (version 4.2.3).

## Supplementary Information

Supplementary Material 1. Table S1: Quantitative mutation features across sample types Table S2: Influence of gene mutations from tumor tissue samples on DFS and OS Table S3: Influence of gene mutations from preoperative plasma samples on DFS and OS Table S4: Influence of gene mutations from postoperative plasma samples on DFS and OS Table S5: Performance of the postoperative ctDNA assay for recurrence Table S6: Gene list of the Geneseeq Prime™ 437-gene panel Table S7: Gene list of 196-gene panel Figure S1: Study cohort and sample analysis overview Figure S2: Quality control metrics for sequencing data Figure S3: Enriched pathway and survival-associated gene overlap. Figure S4: Mutation comparison across sample types and external validation of TNMB staging system.

## Data Availability

The complete targeted DNA-sequencing datasets are available in the Genome Sequence Archive [60] at China's National Genomics Data Center [61], under accession number HRA005335 (GSA-Human), and can be accessed publicly via https://ngdc.cncb.ac.cn/gsa-human. The data that has been deposited and made publicly accessible adheres to the regulations set by the Ministry of Science and Technology of the People's Republic of China. The original sequencing data include individual-specific information and are accessible under controlled conditions. To request access to the data, fill out the application form through the GSA-Human System, and the Data Access Committee will grant it. You can find additional instructions on the GSA-Human System website. All relevant data that support the findings of this study are available upon reasonable request from the corresponding authors (yanghong@sysucc.org.cn).
