# Supplementary material for: Circulating tumor DNA as a marker of molecular residual disease in resected esophageal squamous cell carcinoma
Source: Mol Biomed. 2025 Sep 18;6:65. doi: 10.1186/s43556-025-00310-6 (PMC12443647; doi:10.1186/s43556-025-00310-6)
Supplement: Supplementary file 1 — Supplementary Material 1. Table S1: Quantitative mutation features across sample types Table S2: Influence of gene mutations from tumor tissue samples on DFS and OS Table S3: Influence of gene mutations from preoperative plasma samples on DFS and OS Table S4: Influence of gene mutations from postoperative plasma samples on DFS and OS Table S5: Performance of the postoperative ctDNA assay for recurrence Table S6: Gene list of the Geneseeq Prime™ 437-gene panel Table S7: Gene list of 196-gene panel Figure S1: Study cohort and sample analysis overview Figure S2: Quality control metrics for sequencing data Figure S3: Enriched pathway and survival-associated gene overlap. Figure S4: Mutation comparison across sample types and external validation of TNMB staging system. [file 43556_2025_310_MOESM1_ESM.docx]

**Circulating tumor DNA as a marker of molecular residual disease in resected esophageal squamous cell carcinoma**

Cai-Yan Fang^†^, Jing Wen^†^, Jia-Di Wu^†^, Zhi-Chao Li^†^, Sheng Huang^†^, Yan Huang^†^, Ji-Yang Chen, Hui-Lin Su, Xiu-Ying Xie, Kong-Jia Luo*, Jian-Hua Fu*, and Hong Yang**^*^**

† These authors contributed equally to this work

***Co correspondence:**

Prof. Kongjia Luo, Department of Thoracic Surgery, Sun Yat-Sen University Cancer Center, 651 Dongfeng East Road, Guangzhou, 510060, P. R. China. E-mail address: [luokj@sysucc.org.cn](mailto:luokj@sysucc.org.cn)

**or:** Prof. Jianhua Fu, Department of Thoracic Surgery, Sun Yat-Sen University Cancer Center, 651 Dongfeng East Road, Guangzhou, 510060, P. R. China. E-mail address: [fujh@sysucc.org.cn](mailto:fujh@sysucc.org.cn)

**or:** Prof. Hong Yang, Department of Thoracic Surgery, Sun Yat-Sen University Cancer Center, 651 Dongfeng East Road, Guangzhou, 510060, P. R. China. E-mail address: [yanghong@sysucc.org.cn](mailto:yanghong@sysucc.org.cn)；ORCID: 0000-0002-6007-9086.

**Supplementary material**

Table S1: Quantitative mutation features across sample types

Table S2: Influence of gene mutations from tumor tissue samples on DFS and OS

Table S3: Influence of gene mutations from preoperative plasma samples on DFS and OS

Table S4: Influence of gene mutations from postoperative plasma samples on DFS and OS

Table S5: Performance of the postoperative ctDNA assay for recurrence

Table S6: Gene list of the Geneseeq Prime™ 437-gene panel

Table S7: Gene list of 196-gene panel

Figure S1: Study cohort and sample analysis overview

Figure S2: Quality control metrics for sequencing data

Figure S3: Enriched pathway and survival-associated gene overlap.

Figure S4: Mutation comparison across sample types and external validation of TNMB staging system

**Table S1: Quantitative mutation features across sample types**

| Genomic metric | Tumor tissue (n=62) | Preoperative plasma (n=108) | Postoperative plasma (n=125) |
| --- | --- | --- | --- |
| Total mutations | 455 | 187 | 107 |
| Unique genes | 173 | 63 | 45 |
| Mutations per patient, median(range) | 7(3-21) | 2(1-8) | 2(1-5) |
| Mean VAF | 19.3% | 0.68% | 0.24% |

VAF, mean variant allele frequency

**Table S2: Influence of gene mutations from tumor tissue samples on DFS and OS**

| **Gene** | **incidence** | **Univariate analysis for DFS** | | **Univariate analysis for OS** | |
| --- | --- | --- | --- | --- | --- |
|  |  | **HR（95% CI）** | **p value** | **HR（95% CI）** | **p value** |
| *ATRX* | 1.6% (1/62) | 16.62 (1.84-150.14) | 0.012 |  |  |
| *NFKBIA* | 4.8% (3/62) | 12.11 (3.20-45.89) | <0.001 | 13.72 (3.63 – 51.83) | <0.001 |
| *FANCI* | 4.8% (3/62) | 3.56(1.06-11.97) | 0.040 | 3.80 (1.14 – 12.71) | 0.030 |
| *ATR* | 8.1% (5/62) |  |  | 3.08 (1.16 – 8.18) | 0.024 |

**Table S3: Influence of gene mutations from preoperative plasma samples on DFS and OS**

| **Gene** | **incidence** | **Univariate analysis for DFS** | | **Univariate analysis for OS** | |
| --- | --- | --- | --- | --- | --- |
|  |  | **HR（95% CI）** | **p value** | **HR（95% CI）** | **p value** |
| *TP53* | 56.5% (61/108) | 2.56(1.35-4.86) | 0.004 | 2.06(1.04-4.08) | 0.037 |
| *LRP1B* | 2.8% (3/108) |  |  | 5.26(1.59-17.36) | 0.0065 |
| *SDHA* | 1.9% (2/108) |  |  | 5.52(1.30-23.45) | 0.021 |
| *NFE2L2* | 3.7% (4/108) |  |  | 3.60(1.09-11.81) | 0.035 |

**Table S4: Influence of gene mutations from postoperative plasma samples on DFS and OS**

| **Gene** | **incidence** | **Univariate analysis for DFS** | | **Univariate analysis for OS** | |
| --- | --- | --- | --- | --- | --- |
|  |  | **HR（95% CI）** | **p value** | **HR（95% CI）** | **p value** |
| *CDKN2A* | 3.2% (4/125) | 4.40(1.58-12.29) | 0.0047 |  |  |
| *TP53* | 28.0% (35/125) | 2.20(1.27-3.82) | 0.0049 | 2.24(1.23-4.08) | 0.0086 |
| *TUBB3* | 1.6% (2/125) | 5.51(1.32-23.01) | 0.019 |  |  |
| *SDHA* | 3.2% (4/125) | 3.41(1.21-9.59) | 0.020 | 4.42(1.57-12.44) | 0.0049 |
| *ATM* | 2.4% (3/125) | 3.89(1.20-12.59) | 0.023 |  |  |
| *PIK3CA* | 4.8% (6/125) | 2.75(1.09-6.95) | 0.033 |  |  |
| *VHL* | 0.8% (1/125) | 7.76(1.02-58.72) | 0.047 |  |  |
| *ATR* | 2.4% (3/125) |  |  | 4.44(1.36-14.50) | 0.014 |

**Table S5: Performance of the postoperative ctDNA assay for recurrence**

| Postoperative  ctDNA assay | Within one-year follow-up | | | Within two-year follow-up | | | Within three-year follow-up | | |
| --- | --- | --- | --- | --- | --- | --- | --- | --- | --- |
|  | No Relapse | Relapse | Total | No Relapse | Relapse | Total | No Relapse | Relapse | Total |
| ctDNA-neg | 61 | 4 | 65 | 55 | 10 | 65 | 52 | 13 | 65 |
| ctDNA-pos | 40 | 20 | 60 | 24 | 36 | 60 | 22 | 38 | 60 |
| Total | 101 | 24 | 125 | 79 | 46 | 125 | 74 | 51 | 125 |
| Sensitivity | 83.33% (62.62%-95.26%) | | | 78.26% (63.64%-89.05%) | | | 74.51% (60.37%-85.67%) | | |
| Specificity | 60.40% (50.17%-69.99%) | | | 69.62% (58.25%-79.47%) | | | 70.27% (58.52%-80.34%) | | |
| PPV | 33.33% (21.69%-46.69%) | | | 60.00% (46.54%-72.44%) | | | 63.33% (49.90%-75.41%) | | |
| NPV | 93.85% (84.99%-98.30%) | | | 84.62% (73.52%-92.37%) | | | 80.00% (68.23%-88.90%) | | |

PPV, positive predictive value; NPV, negative predictive value

**Table S6: Gene list of the Geneseeq Prime™ 437-gene panel.**

| **Gene list of the Geneseeq Prime™ 437-gene panel** | | | | | | | | | |
| --- | --- | --- | --- | --- | --- | --- | --- | --- | --- |
| *ABCB1 (MDR1)* | *BRCA2* | *CYP19A1* | *ETV5* | *GRM3* | *KMT2D (MLL2)* | *NCOR1* | *PMS2* | *RICTOR* | *TAP2* |
| *ABCC2 (MRP2)* | *BRD4* | *CYP2A13* | *ETV6* | *GRM8* | *KRAS* | *NF1* | *POLD1* | *RNF43* | *TEK* |
| *ADGRB3 (BAI3)* | *BRIP1* | *CYP2A6* | *EWSR1* | *GSTM1* | *LHCGR* | *NF2* | *POLD3* | *ROS1* | *TEKT4* |
| *ADH1B* | *BTG2* | *CYP2A7* | *EXT1* | *GSTM4* | *LMO1* | *NFE2L2* | *POLE* | *RPTOR* | *TERC* |
| *AFDN (MLLT4)* | *BTK* | *CYP2B6*6* | *EXT2* | *GSTP1* | *LRP1B* | *NFKBIA* | *POLH* | *RRM1* | *TERT* |
| *AIP* | *BUB1B* | *CYP2C19*2* | *EZH2* | *GSTT1* | *LYN* | *NKX2-1* | *POT1* | *RUNX1* | *TET2* |
| *AKT1* | *CASP8* | *CYP2C9*3* | *EZR* | *HDAC2* | *LZTR1* | *NOTCH1* | *PPARD* | *RUNX1T1* | *TGFBR2* |
| *AKT2* | *CBL* | *CYP2D6* | *FANCA* | *HDAC9* | *MAP2K1 (MEK1)* | *NOTCH2* | *PPM1D* | *SBDS* | *THADA* |
| *AKT3* | *CBLB* | *CYP3A4*4* | *FANCC* | *HGF* | *MAP2K2 (MEK2)* | *NOTCH3* | *PPP2R1A* | *SDC4* | *TMEM127* |
| *ALDH2* | *CCN6 (WISP3)* | *CYP3A5* | *FANCD2* | *HLA-A* | *MAP2K4* | *NPM1* | *PPP2R2A* | *SDHA* | *TMPRSS2* |
| *ALK* | *CCND1* | *CYSLTR2* | *FANCE* | *HNF1A* | *MAP3K1* | *NQO1* | *PRDM1* | *SDHB* | *TNFAIP3* |
| *AMER1 (FAM123B)* | *CCNE1* | *DAXX* | *FANCF* | *HNF1B* | *MAP3K4* | *NRAS* | *PREX2* | *SDHC* | *TNFRSF11A* |
| *APC* | *CD274 (PD-L1)* | *DDR2* | *FANCG* | *HOXB13* | *MAX* | *NRG1* | *PRF1* | *SDHD* | *TNFRSF14* |
| *AR* | *CD74* | *DENND1A* | *FANCI* | *HRAS* | *MC1R* | *NSD1* | *PRKACA* | *SEPTIN9 (SEPT9)* | *TNFRSF19* |
| *ARAF* | *CDA* | *DHFR* | *FANCL* | *IDH1* | *MCL1* | *NTHL1* | *PRKAR1A* | *SETBP1* | *TNFSF11* |
| *ARID1A* | *CDC73* | *DICER1* | *FANCM* | *IDH2* | *MDM2* | *NTRK1* | *PRKCI* | *SETD2* | *TOP1* |
| *ARID1B* | *CDH1* | *DLL3* | *FAT1* | *IFNA6* | *MDM4* | *NTRK2* | *PRKDC* | *SF3B1* | *TOP2A* |
| *ARID2* | *CDK10* | *DNMT3A* | *FBXW7* | *IFNB1* | *MECOM* | *NTRK3* | *PRKN (PARK2)* | *SGK1* | *TP53* |
| *ARID5B* | *CDK12* | *DOT1L* | *FGF19* | *IFNE* | *MED12* | *NUTM1* | *PRSS1* | *SKP2* | *TP63* |
| *ASCL4* | *CDK4* | *DPYD* | *FGFR1* | *IFNG* | *MEF2B* | *PAK3* | *PRSS3* | *SLC34A2* | *TPMT* |
| *ASXL1* | *CDK6* | *DTL(CDT2)* | *FGFR2* | *IFNGR1* | *MEN1* | *PALB2* | *PTCH1* | *SLC3A2* | *TSC1* |
| *ATF1* | *CDK8* | *DUSP2* | *FGFR3* | *IFNGR2* | *MET* | *PALLD* | *PTEN* | *SMAD2* | *TSC2* |
| *ATIC* | *CDKN1A* | *EGFR* | *FGFR4* | *IGF1R* | *MGMT* | *PARP1* | *PTK2* | *SMAD3* | *TSHR* |
| *ATM* | *CDKN1B* | *EIF1AX* | *FH* | *IGF2* | *MITF* | *PARP2* | *PTPN11* | *SMAD4* | *TTF1* |
| *ATR* | *CDKN1C* | *EML4* | *FLCN* | *IKBKE* | *MLH1* | *PAX5* | *PTPN13* | *SMAD7* | *TUBB3* |
| *ATRX* | *CDKN2A* | *EMSY (c11orf30)* | *FLT1 (VEGFR1)* | *IKZF1* | *MLH3* | *PBRM1* | *QKI* | *SMARCA4* | *TYMS* |
| *AURKA* | *CDKN2B* | *EP300* | *FLT3* | *IL7R* | *MLLT1* | *PDCD1 (PD1)* | *RAC1* | *SMARCB1* | *U2AF1* |
| *AURKB* | *CDKN2C* | *EPAS1* | *FLT4* | *INPP4B* | *MLLT3* | *PDCD1LG2 (PD-L2)* | *RAC3* | *SMO* | *UGT1A1* |
| *AXIN2* | *CEBPA* | *EPCAM* | *FOXA1* | *IRF2* | *MPL* | *PDE11A* | *RAD50* | *SOCS1* | *VAMP2* |
| *AXL* | *CEP57* | *EPHA2* | *FOXL2* | *JAK1* | *MRE11 (MRE11A)* | *PDGFRA* | *RAD51* | *SOS1* | *VEGFA* |
| *B2M* | *CHD4* | *EPHA3* | *FOXO1* | *JAK2* | *MSH2* | *PDGFRB* | *RAD51B* | *SOX2* | *VHL* |
| *BAD* | *CHD8* | *EPHA5* | *FOXP1* | *JAK3* | *MSH6* | *PDK1* | *RAD51C* | *SPOP* | *WAS* |
| *BAK1* | *CHEK1* | *ERBB2 (HER2)* | *FRG1* | *JARID2* | *MTAP* | *PGR* | *RAD51D* | *SPRED1* | *WRN* |
| *BAP1* | *CHEK2* | *ERBB3* | *GATA1* | *JUN* | *MTHFR* | *PHOX2B* | *RAD54L* | *SPRY4* | *WT1* |
| *BARD1* | *CREBBP* | *ERBB4* | *GATA2* | *KDM5A* | *MTOR* | *PIK3C3* | *RAF1* | *SRC* | *XPA* |
| *BAX* | *CRKL* | *ERBIN (ERBB2IP)* | *GATA3* | *KDR (VEGFR2)* | *MUTYH* | *PIK3CA* | *RARA* | *SRSF2* | *XPC* |
| *BCL2* | *CSF1R* | *ERCC1* | *GATA4* | *KEAP1* | *MYBL1* | *PIK3CD* | *RARG* | *SRY* | *XRCC1* |
| *BCL2L11 (BIM)* | *CTCF* | *ERCC2* | *GATA6* | *KIF1B* | *MYC* | *PIK3R1* | *RASGEF1A* | *STAG2* | *XRCC2* |
| *BCR* | *CTLA4* | *ERCC3* | *GNA11* | *KIT* | *MYCL (MYCL1)* | *PIK3R2* | *RB1* | *STAT3* | *YAP1* |
| *BIRC3* | *CTNNB1* | *ERCC4* | *GNAQ* | *KITLG* | *MYCN* | *PKHD1* | *RECQL4* | *STK11* | *ZNF217* |
| *BLM* | *CUL3* | *ERCC5* | *GNAS* | *KLLN* | *MYD88* | *PLAG1* | *RELN* | *STMN1* | *ZNF703* |
| *BMPR1A* | *CUX1* | *ESR1* | *GREB1* | *KMT2A (MLL)* | *MYH9* | *PLCB4* | *RET* | *SUFU* |  |
| *BRAF* | *CXCR4* | *ETV1* | *GREM1* | *KMT2B* | *NAT1* | *PLK1* | *RHBDF2* | *TACC3* |  |
| *BRCA1* | *CYLD* | *ETV4* | *GRIN2A* | *KMT2C* | *NBN* | *PMS1* | *RHOA* | *TAP1* |  |

**Table S7: Gene list of 196-gene panel**

| **Gene list of the 196-gene panel** | | | | | | | | | | |
| --- | --- | --- | --- | --- | --- | --- | --- | --- | --- | --- |
| *AKT1* | *BRIP1* | *CYP2C19* | *ESR1* | *HDAC9* | *MECOM* | *NTRK1* | *PTPN11* | *SDHC* | *THADA* |  |
| *AKT2* | *BTK* | *CYP2D6* | *FANCA* | *HGF* | *MED12* | *NTRK2* | *PTPRD* | *SDHD* | *TOP1* |  |
| *AKT3* | *CBL* | *CYP3A4* | *FANCC* | *HRAS* | *MET* | *NTRK3* | *RAD51* | *SETD2* | *TOP2A* |  |
| *ALK* | *CD274 (PD-L1)* | *CYP3A5* | *FANCD2* | *IDH1* | *MGMT* | *PALB2* | *RAD51B* | *SF3B1* | *TP53* |  |
| *APC* | *CD74* | *DDR2* | *FANCI* | *IDH2* | *MLH1* | *PBRM1* | *RAD51C* | *SGK1* | *TPMT* |  |
| *AR* | *CDA* | *DNMT3A* | *FANCL* | *INPP4B* | *MLH3* | *PDCD1(PD1)* | *RAD51D* | *SLC34A2* | *TSC1* |  |
| *ARAF* | *CDH1* | *DPYD* | *FAT1* | *JAK1* | *MRE11* | *PDCD1LG2(PD-L2)* | *RAD54L* | *SMAD2* | *TSC2* |  |
| *ARID1A* | *CDK12* | *DHFR* | *FBXW7* | *JAK2* | *MSH2* | *PDGFRA* | *RAF1* | *SMAD3* | *TUBB3* |  |
| *ARID2* | *CDK4* | *EGFR* | *FGFR1* | *KDM6A* | *MSH6* | *PDGFRB* | *RB1* | *SMAD4* | *TYMS* |  |
| *ASXL1* | *CDK6* | *EML4* | *FGFR2* | *KDR(VEGFR2)* | *MTHFR* | *PIK3CA* | *RET* | *SMARCA4* | *U2AF1* |  |
| *ATM* | *CDK8* | *EP300* | *FGFR3* | *KEAP1* | *MTOR* | *PIK3CD* | *RHBDF2* | *SMARCB1* | *UGT1A1* |  |
| *ATR* | *CDKN1B* | *EPCAM* | *FH* | *KIT* | *MUTYH* | *PIK3R1* | *RHOA* | *SOX2* | *VEGFA* |  |
| *ATRX* | *CDKN2A* | *EPHA3* | *FLT4* | *KMT2A (MLL)* | *MYC* | *PMS1* | *RICTOR* | *SRY* | *VHL* |  |
| *AXL* | *CDKN2B* | *ERBB2 (HER2)* | *GATA4* | *KMT2D (MLL2)* | *NBN* | *PMS2* | *RNF43* | *STAG2* | *WRN* |  |
| *BARD1* | *CHEK1* | *ERBB3* | *GNAQ* | *KRAS* | *NF1* | *POLD1* | *ROS1* | *STAT3* | *XRCC1* |  |
| *BCL2L11(BIM)* | *CHEK2* | *ERBB4* | *GNAS* | *LRP1B* | *NF2* | *POLE* | *RRM1* | *STK11* | *YAP1* |  |
| *BLM* | *CREBBP* | *ERCC1* | *GRIN2A* | *LZTR1* | *NFE2L2* | *PPP2R2A* | *RUNX1T1* | *STMN1* |  |  |
| *BRAF* | *CTNNB1* | *ERCC2* | *GSTM1* | *MAP2K1 (MEK1)* | *NOTCH1* | *PRKCI* | *SDC4* | *TERT* |  |  |
| *BRCA1* | *CYP2A6* | *ERCC3* | *GSTP1* | *MAP2K2 (MEK2)* | *NQO1* | *PTCH1* | *SDHA* | *TET2* |  |  |
| *BRCA2* | *CYP2B6* | *ERCC4* | *GSTT1* | *MAP4K3* | *NRAS* | *PTEN* | *SDHB* | *TGFBR2* |  |  |

**
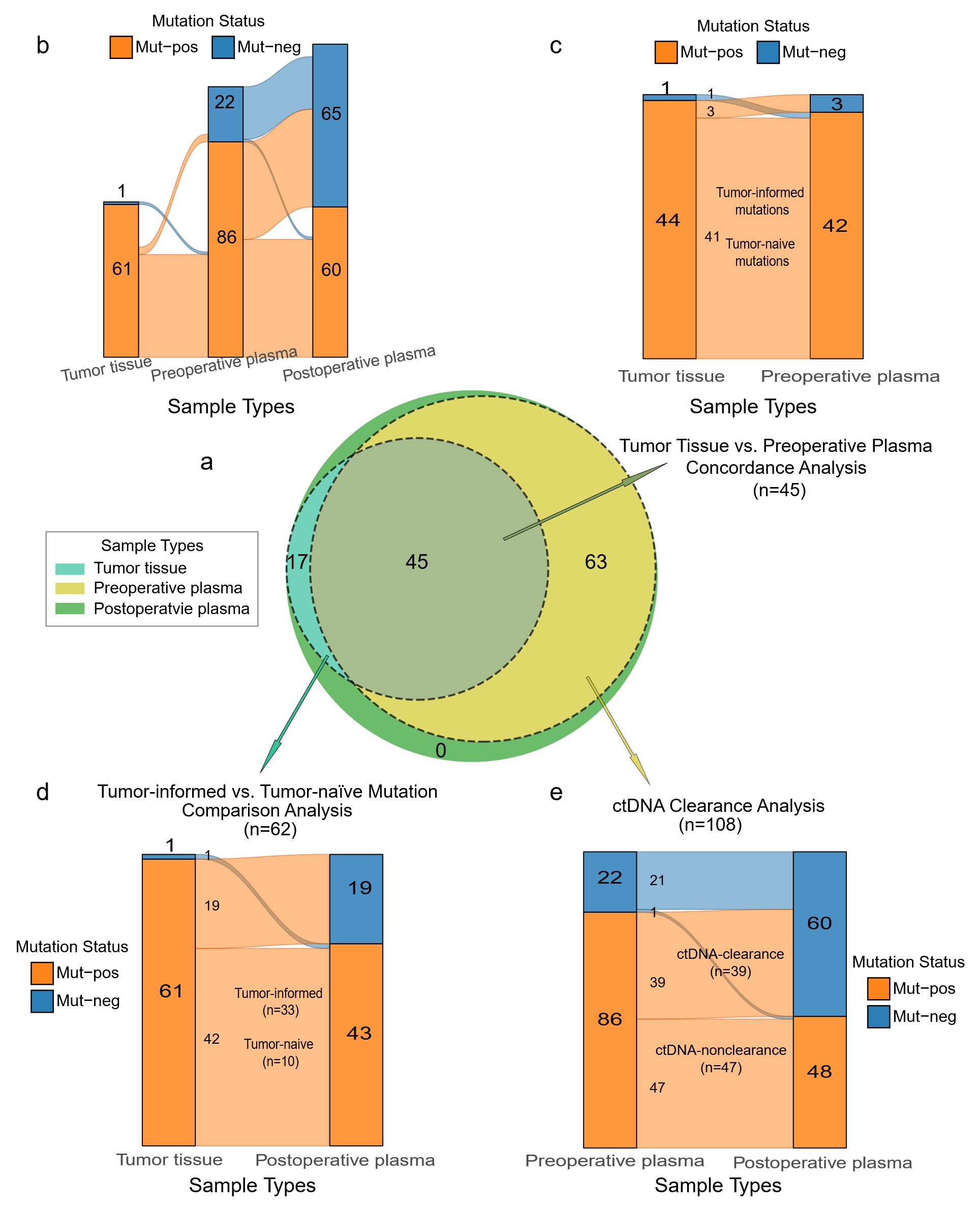
**

**Figure S1. Study cohort and sample analysis overview. a.** Distribution of matched sample types among 125 patients, showing pairings between tumor tissue, preoperative plasma, and postoperative plasma specimens. **b.** Mutation status distribution among the three sample types in the entire cohort (n=125). **c.** Mutation status of patients with paired tumor tissue and preoperative plasma samples (n=45), used for concordance analysis. **d.** Mutation status of patients with paired tumor tissue and postoperative plasma samples (n=62), comparing tumor-informed versus tumor-naïve mutations. **e.** Mutation status of patients with paired preoperative and postoperative plasma samples (n=108), analyzed for ctDNA clearance. Mut-neg, mutation-negative; Mut-pos, mutation-positive.

**
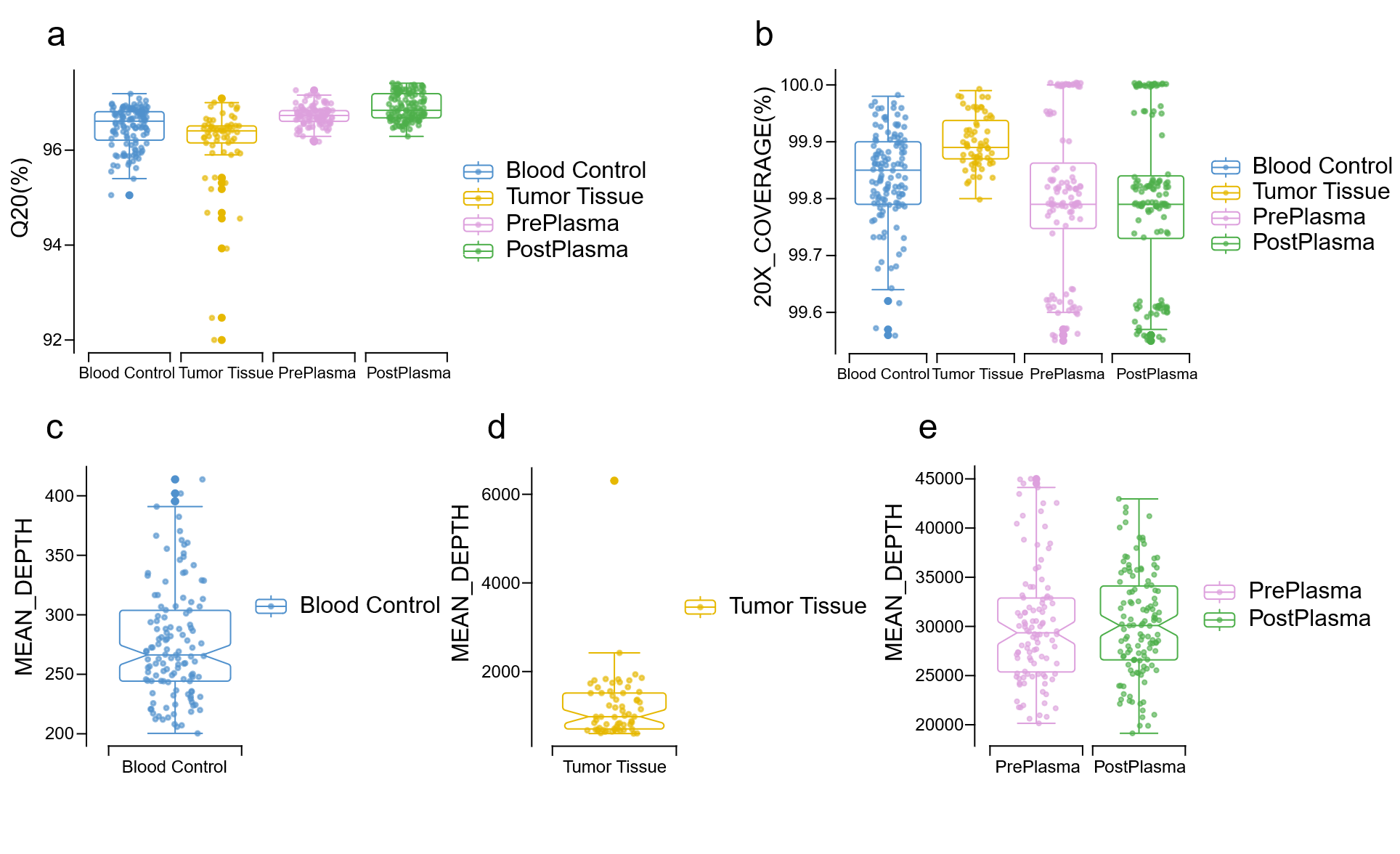
Figure S2** **Quality control metrics for sequencing data a-b.** Q20 (a) and 20X coverage rate (b) across blood control, tumor tissue, preoperative plasma, and postoperative plasma samples. **c-e.** Mean sequencing depths for the four sample types.


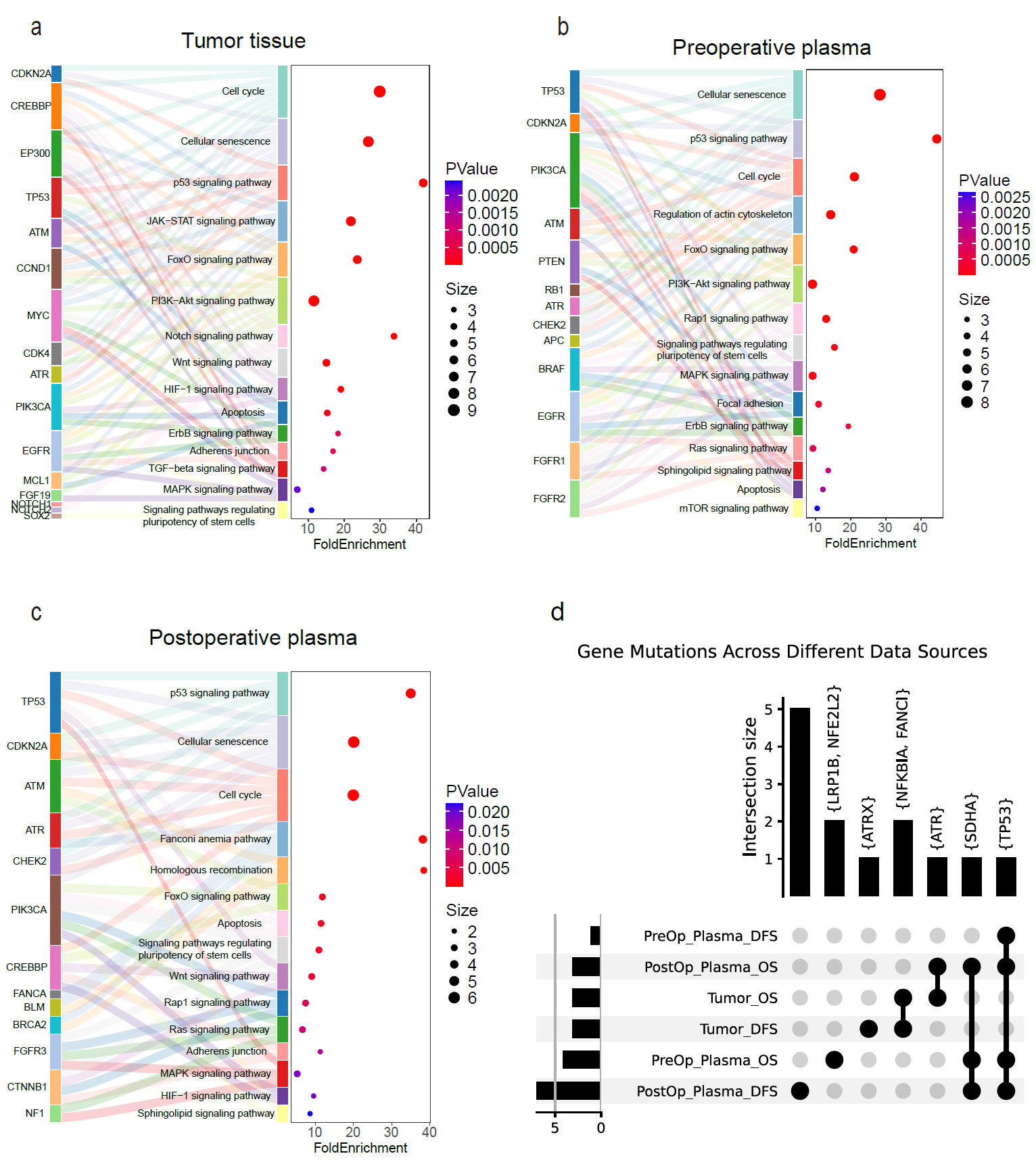


**Figure S3** **Enriched pathway and survival-associated gene overlap. a-c.** Sanger plots and lollipop diagrams display the top 15 KEGG pathways (ranked by p-value) for the 20 most frequently mutated genes in: (a) tumor tissue, (b) preoperative plasma, and (c) postoperative plasma samples. **d.** UpSet plot illustrating overlapping mutated genes associated with survival across sample types.

**
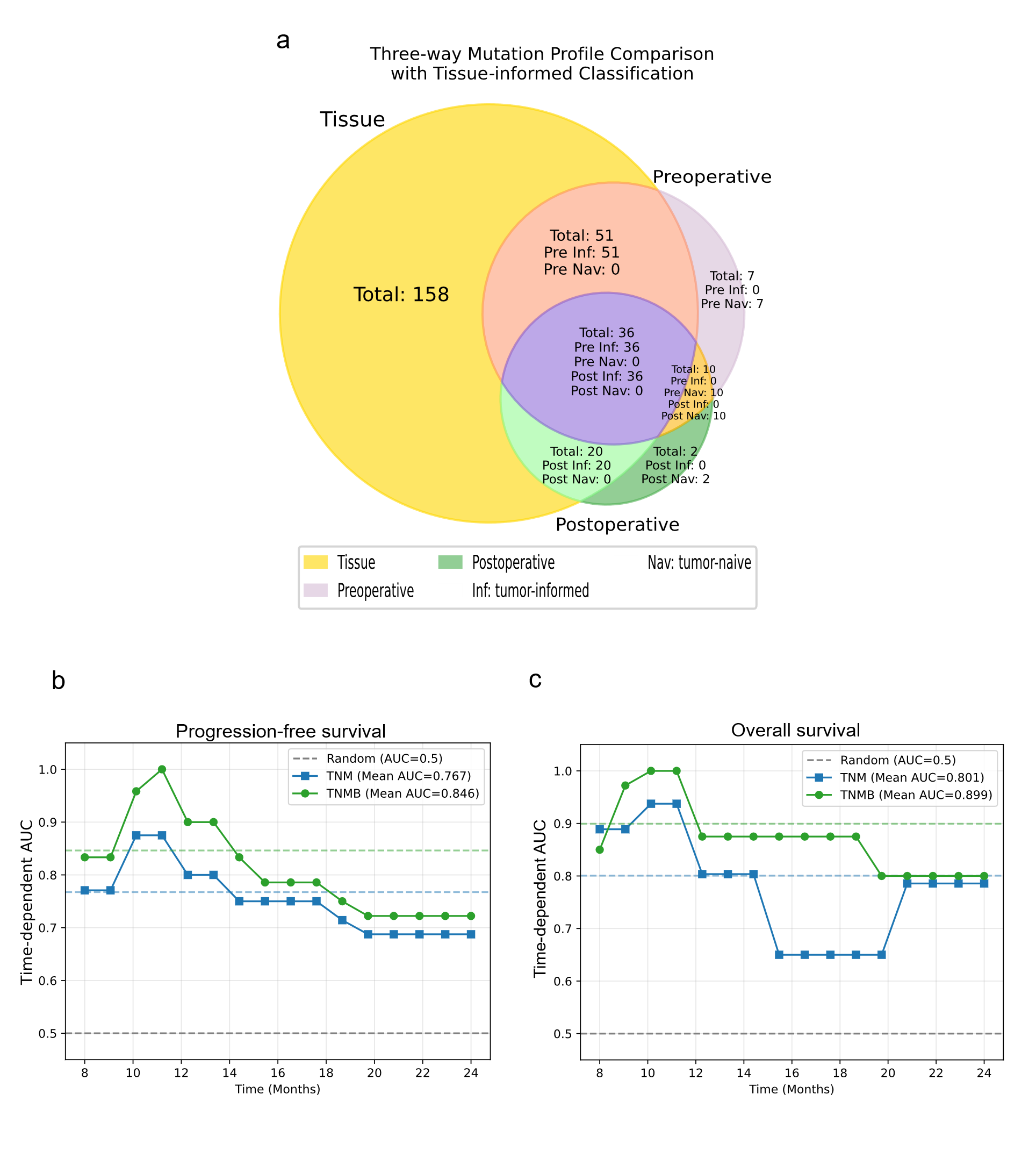
**

**Figure S4** **Mutation comparison across sample types and external validation of TNMB staging** **system** **a.** Venn diagram of mutation concordance across matched tumor tissue, preoperative plasma, and postoperative plasma (n = 45 patients). **b-c.** External validation in ESCC patients receiving chemoradiotherapy plus toripalimab confirms TNMB's superior prognostic performance over TNM staging, as evidenced by time-dependent ROC curves for (b) PFS (C-index: 0.75 vs. 0.71) and (c) OS (C-index: 0.80 vs. 0.72). PFS, progression-free survival; OS, overall survival.
